# SMURF1 silencing diminishes a CD44-high cancer stem cell-like population in head and neck squamous cell carcinoma

**DOI:** 10.1186/1476-4598-13-260

**Published:** 2014-12-03

**Authors:** Ali Khammanivong, Raj Gopalakrishnan, Erin B Dickerson

**Affiliations:** Department of Veterinary Clinical Sciences, College of Veterinary Medicine, University of Minnesota, 1352 Boyd Avenue, Saint Paul, MN 55108 USA; Department of Diagnostic and Biological Sciences, School of Dentistry, University of Minnesota, 515 Delaware Street SE, Minneapolis, MN 55455 USA; Masonic Cancer Center, University of Minnesota, 425 East River Parkway, Minneapolis, MN 55455 USA

**Keywords:** BMP signaling, SMURF1, Cancer stem cell, HNSCC, CD44, pSMAD1/5/8

## Abstract

**Background:**

Bone morphogenetic protein (BMP) signaling is thought to play key roles in regulating the survival and maintenance of cancer stem cells (CSCs), which contribute to disease recurrences and treatment failures in many malignances, including head and neck squamous cell carcinoma (HNSCC). Intracellular BMP signaling is regulated by SMAD specific E3 ubiquitin protein ligase 1 (SMURF1) during cellular development. However, little is known about the role or regulation of BMP signaling in HNSCC CSCs.

**Methods:**

Two CSC-like populations, CD44^high^/BMI1^high^ and CD44^high^/ALDH^high^, were enriched from HNSCC cell lines and evaluated for the expression of SMURF1 by qRT-PCR, flow cytometry, and immunoblotting. The activation status of BMP signaling in these populations was determined by using immunoblotting to detect phosphorylated SMAD1/5/8 (pSMAD1/5/8) levels. Knockdown of SMURF1 transcripts by RNA interference was used to assess the role of SMURF1 in BMP signaling and CSC maintenance. Loss of CSC-like phenotypes following SMURF1 knockdown was determined by changes in CD44^high^ levels, cellular differentiation, and reduction in colony formation.

**Results:**

Populations of enriched CSC-like cells displayed decreased levels of pSMAD1/5/8 and BMP signaling target gene ID1 while SMURF1, CD44, and BMI1 were highly expressed when compared to non-CSC populations. Stable knockdown of SMURF1 expression in CSC-like cells increased pSMAD1/5/8 protein levels, indicating the reactivation of BMP signaling pathways. Decreased expression of SMURF1 also promoted adipogenic differentiation and reduced colony formation in a three-dimensional culture assay, indicating loss of tumorigenic capacity. The role of SMURF1 and inhibition of BMP signaling in maintaining a CSC-like population was confirmed by the loss of a CD44^high^ expressing subpopulation in SMURF1 knockdown cells.

**Conclusions:**

Our findings suggest that inhibition of BMP signaling potentiates the long-term survival of HNSCC CSCs, and that this inhibition is mediated by SMURF1. Targeting SMURF1 and restoring BMP signaling may offer a new therapeutic approach to promote differentiation and reduction of CSC populations leading to reduced drug resistance and disease recurrence.

**Electronic supplementary material:**

The online version of this article (doi:10.1186/1476-4598-13-260) contains supplementary material, which is available to authorized users.

## Background

Head and neck squamous cell carcinoma (HNSCC) is among the most prevalent cancers worldwide, with approximately 500,000 incidences per year, including close to 40,000 new cases in the United States [1–3]. Despite the successes of standard treatment modalities such as surgery, radiation, and chemotherapies for the majority of patients with early stage disease [4, 5], patients with regional and distant metastases or recurrent disease make up a substantial proportion of the treatment failures [2, 3, 6], bringing the 5-year survival rate to under 50%. Thus, the development of chemoresistance and metastatic disease remains a continuing challenge for HNSCC patients, and overcoming these obstacles requires an improved understanding of the underlying mechanisms contributing to tumorigenesis and drug resistance.

Evidence suggests that a subpopulation of cells, known as cancer stem cells (CSCs), possess the potential for self-renewal, multipotent differentiation, and tumorigenesis [5, 7, 8], and these cells may contribute to the aggression and dismal prognosis of HNSCC. Although chemo- and radiotherapy eliminate many of the bulk tumor cells, CSCs possess traits that allow them to survive and repopulate. The surviving cells can then regenerate tumors leading to disease recurrences that are less responsive to conventional therapies. In HNSCC, CSC populations were first identified using the cell surface marker CD44 [9]. A subset of CD44^high^ expressing cells was shown to possess stem cell properties along with the ability to initiate tumorigenesis in mice compared to CD44^low/-^ cells [9–11]. Aldehyde dehydrogenase (ALDH) activity also has been used to identify CSCs in HNSCC [12–14]. ALDH activity correlates strongly with increased resistance to chemo- and radiotherapy [15, 16], and the combination of ALDH activity with CD44 expression is more selective for CSC-like populations than either marker alone [17–20].

While it is established that the implantation of extremely small numbers of ALDH^high^/CD44^high^ cells consistently give rise to tumors *in vivo* further solidifying their tumorigenic properties [19, 21], it remains relatively unclear how the expression of ALDH and CD44 are regulated in these populations. For ALDH, the epithelial-to-mesenchymal transition regulator Snail was found to be a key factor in maintaining the CSC properties in HNSCC. Knockdown of Snail decreased ALDH expression, inhibited CSC-like properties, and attenuated tumorigenesis in ALDH^high^/CD44^high^ cells [12]. While factors regulating CD44 expression in HNSCC are unknown, clues may come from studies in chondrocytes where co-immunoprecipitation experiments identified the interaction of SMAD1 with CD44. The interaction of SMAD1 with CD44 provides a link between CD44 and the bone morphogenetic (BMP) signaling cascade, which signals through a family of SMAD proteins [22]. The SMAD1/CD44 interaction appears to sequester SMAD1 in the cytoplasm, but the nuclear accumulation of SMAD1 increases upon BMP7 stimulation [23]. The SMAD1/CD44 interaction also is associated with reversible dormancy of CSCs along with the potential for tumor recurrence and metastasis in prostate cancer [24]. Thus, BMP signaling through SMAD proteins may be important for regulating and maintaining HNSCC CSCs and in the overall regulation of CD44 expression and signaling.

BMPs are members of the transforming growth factor beta (TGF-β) superfamily with diverse biological functions, including regulation of embryogenesis, cell proliferation, migration, differentiation, and apoptosis [25–28]. Extracellular regulation of BMP signaling is tightly regulated by factors such as noggin (NOG), chordin (CHRD), and twisted gastrulation BMP signaling modulator 1 (TWSG1) [29, 30]. Intracellular regulation is primarily mediated by SMAD-specific E3 ubiquitin ligase 1 (SMURF1) through its interactions with SMADs. Recently, ubiquitin ligases have emerged as critical regulators for the development and function of stem cell and stem cell-like populations. For example, the E3 ligases Itch and c-Cbl have been identified as regulators of hematopoietic stem cell homeostasis and function [31, 32]. In glioblastoma, two isoforms of the protein Numb differentially interacted with the SCF^Fbw7^ ubiquitin ligase assembly to regulate the glioblastoma cancer stem cell hierarchy [33]. Based on these findings, it is likely that other E3 ligases play similar roles in other cancers. This prompted us to investigate the role of SMURF1 in the regulation of BMP signaling and in the maintenance of HNSCC CSCs.

In this study, we investigated whether the E3 ligase SMURF1 is involved in regulating BMP signaling and the maintenance of CD44^high^ cells in head and neck cancer cell lines. We demonstrated that cell lines grown under non-adherent culture conditions or isolated from ALDH^high^/CD44^high^ populations showed inhibition of BMP signaling. Silencing of SMURF1 expression increased BMP signaling and reduced the abundance of the CD44^high^ population in HNSCC cell lines. The inverse relationship between SMURF1 expression and BMP signaling indicates that BMP signaling may be important in regulating CD44 expression and, in turn, regulating CSC populations in HNSCC. Reducing SMURF1 activity to enhance BMP signaling may limit CSC function in HNSCC and provide new therapeutic approaches for reducing drug resistant populations.

## Results

### Sphere cells generated from HNSCC cell lines show CSC-like characteristics

**Figure 1:**
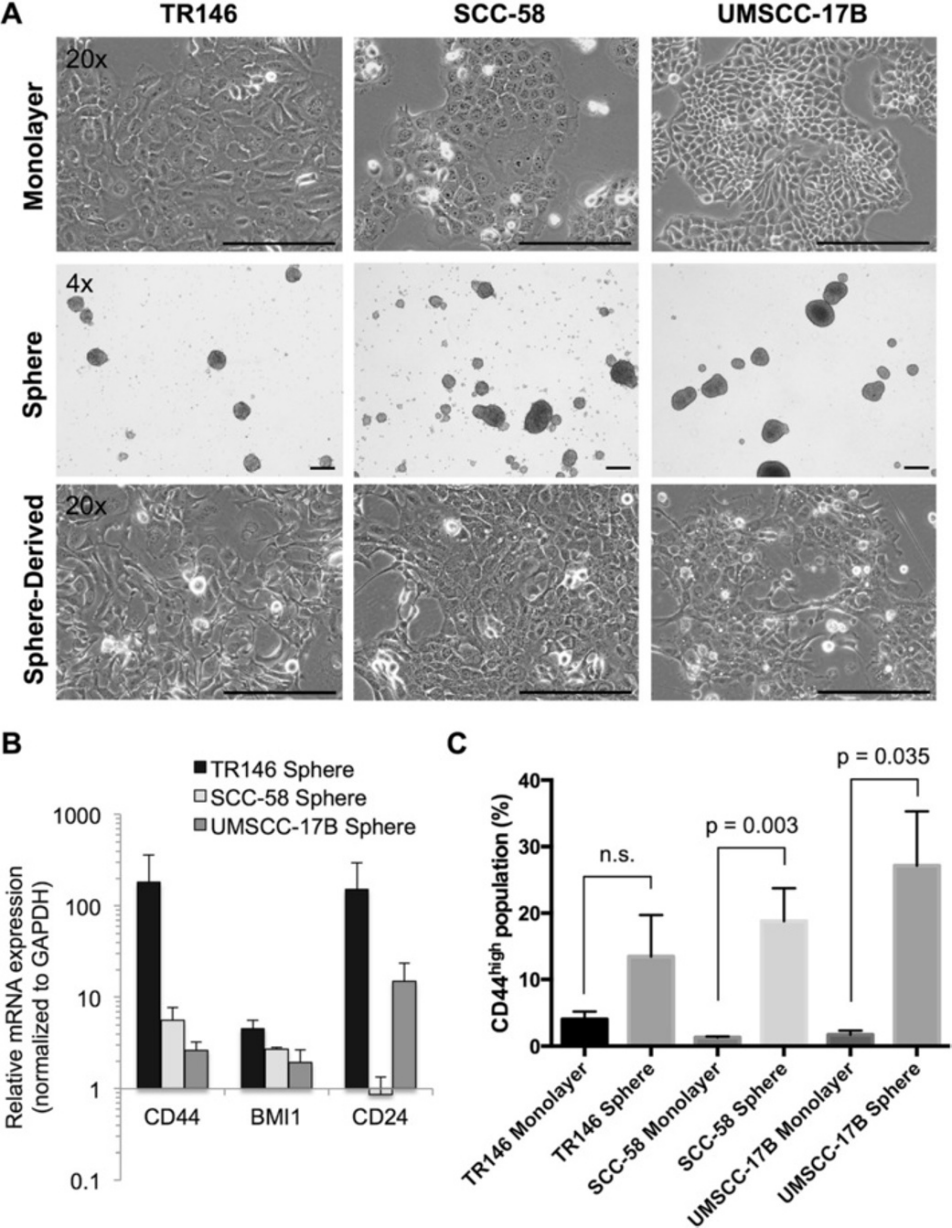
**Sphere cells from HNSCC cell lines show CSC-like characteristics. (A)** HNSCC cell lines grown as monolayers with 10% FBS under standard adherent growth conditions (top), as spheres in serum-free suspension growth conditions (middle), and as sphere-derived cells (SDCs) under adherent growth conditions in the presence of 10% FBS and 75 μg/mL Matrigel (bottom). Images were taken using phase contrast microscopy at 20x objective for monolayer and SDCs, 4x for sphere cultures. Scale bars represent 100 μm in length. **(B)** Expression of CSC markers CD44 and BMI1 and differentiation marker CD24 in sphere cells relative to monolayer cultures by qRT-PCR. Expression was normalized to the GAPDH housekeeping gene as an internal control and shown as mean fold change ± SE (n ≥ 3). **(C)** Summary of flow cytometry analysis of CD44 surface protein expression showing the percentages of the CD44^high^ populations in monolayer and sphere cells from TR146 (n = 8), SCC-58 (n = 8) and UMSCC-17B (n = 3). Representative flow cytometry data showing the gating of the CD44^high^ cells are shown in Additional file 1: Figure S1. Data are presented as mean ± SE for the indicated total number of independent experiments. Statistical analysis was performed using two-tailed Student’s *t-test* with equal variance. n.s. = not significant (p > 0.05).

To study the role of BMP signaling in modulating CSC populations, we established stable sphere cultures to enrich for CSC-like cells from three HNSCC cell lines, TR146, SCC-58, and UMSCC-17B. These lines were chosen for their ability to form spheres, along with long-term survival and sphere regeneration following multiple passages in cell culture (Figure 1A). Sphere formation in serum-free suspension cultures has been well established as a means to enrich for cells with CSC-like properties, including drug resistance, self-renewal capability, high expression of stem cell markers (e.g., CD44, ALDH and BMI1), tumor initiation, and differentiation capacity [10, 34–36].

To establish our system, we first sought to demonstrate that sphere cells derived from the three cell lines possessed properties previously attributed to HNSCC CSCs. To do this, we utilized single cell suspensions of sphere cells or sphere cells that were able to re-establish adherent monolayer cultures in the presence of Matrigel, generating populations of sphere-derived cells or SDCs. CSC-like cells grown as spheres in culture are highly quiescent and divide slowly, limiting cell numbers and analysis, so we used SDCs in some studies to partially circumvent this issue. Sphere cells or SDCs grown for 18–24 hours on Matrigel showed increased expression of CD44 and BMI1 (Figure 1B) along with an increase in the percentage of cells in the CD44^high^ population (Figure 1C). BMI1 and CD44^high^ are two reported markers for HNSCC CSCs [9, 37, 38]. Representative flow cytometry analysis of how the CD44^high^ populations were measured in each cell line is shown in supplementary Additional file 1: Figure S1. CD24 expression, reported to be variable in HNSCC CSCs [39], was upregulated in TR146 and UMSCC-17B sphere cells (Figure 1B), but its expression remained unchanged in SCC-58 sphere cells. Sphere cells were highly resistant to cisplatin compared to their monolayer cultures (Additional file 2: Figure S2), and ALDH activity was either unchanged or slightly reduced when compared to those from cells grown as adherent monolayers (data not shown). Because CD44 and BMI1 expression were consistently expressed in sphere cells and SDCs from the three cell lines, sphere cells grown under non-adherent conditions for 10 to 14 days were used as representative CSC-like populations, and all SDCs were derived from these populations for the remaining studies.

### BMP signaling and SMURF1 are differentially regulated in sphere cells

**Figure 2:**
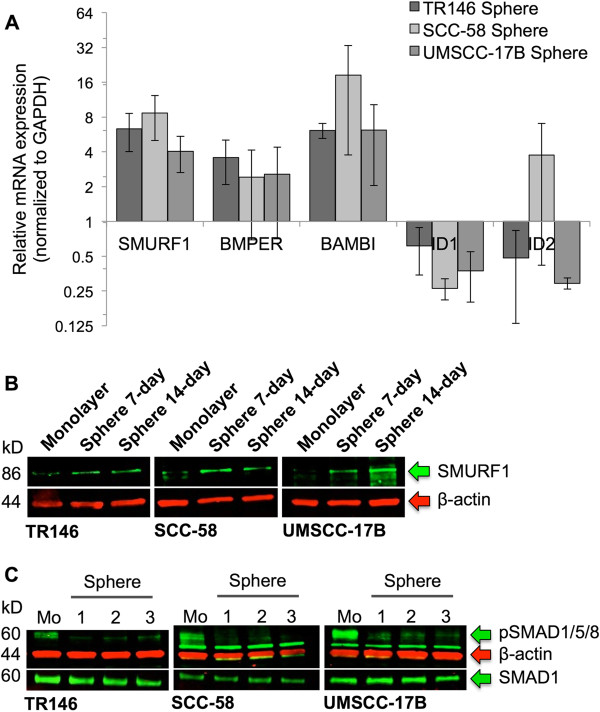
**BMP signaling and SMURF1 are differentially regulated in HNSCC CSC-like sphere cells. (A)** Summary of qRT-PCR array data showing relative expression of BMP signaling inhibitors (SMURF1, BMPER, and BAMBI) and BMP signaling target genes ID1 and ID2 in sphere cell cultures compared to the monolayer cell cultures. Data are shown as mean ± SE with n ≥ 3. **(B)** Immunoblotting of SMURF1 in monolayer cells and spheres cells from 7-day and 14-day sphere cell culture, shown as a representative of at least three independent repeats. **(C)** Immunoblotting of pSMAD1/5/8 and total SMAD1 in monolayer (Mo) and three independent, 14-day sphere cultures. β-actin was used as total protein loading control for all samples.

To determine if BMP signaling pathways were differentially regulated in sphere versus monolayer cells, we compared the gene expression profiles of BMP signaling molecules using qRT-PCR arrays. The BMP signaling inhibitors SMURF1, BMPER, and BAMBI were upregulated in all sphere cell populations while the BMP signaling target genes ID1 and ID2 were downregulated (Figure 2A). However, BMPER and ID2 expression was not consistent and varied between the three cell lines. Because SMURF1 has been shown to inhibit mesenchymal stem cell proliferation and differentiation [40] and to promote invasiveness and CSC properties in pancreatic cancer [41], we chose to validate its expression at the protein level and determine if BMP signaling was inhibited in the sphere cells. We confirmed that SMURF1 protein expression was increased in sphere cells grown in cell culture for 7 days when compared to SMURF1 levels in monolayer cells, and that this increase persisted in sphere cells through day 14 (Figure 2B). SMURF1 expression in monolayer cells was low or not detected. We then measured phosphorylated SMAD1, 5 and 8 (pSMAD1/5/8) levels to determine the status of BMP signaling in the sphere cells. Phosphorylated SMAD1/5/8 proteins are indicative of active BMP signaling; therefore decreased pSMAD1/5/8 levels indicate inhibition of this signaling pathway. Using immunoblotting, we determined that the pSMAD1/5/8 levels in spheres were greatly reduced when compared to those of cultured monolayer cells (Figure 2C), suggesting that BMP signaling was inhibited in sphere cells but active in monolayers. Reduced BMP signaling appeared to be specific for the canonical BMP signaling pathway since phosphorylated p38 MAPK levels, a downstream marker of non-canonical BMP signaling, did not differ between the sphere and monolayer cell populations (data not shown).

Because the reduced levels of pSMAD1/5/8 could be attributed to a loss or reduction of BMP receptor ligand expression rather than inhibition of the pathway by SMURF1, we measured BMP2, 4, and 7 expression by qRT-PCR and secretion of BMP2 by ELISA (Additional file 3: Figure S3). These BMPs activate the BMP signaling pathway by increasing pSMAD1/5/8 phosphorylation critical for cellular growth and differentiation [25, 42, 43]. The sphere cells showed increased expression of BMP2 and BMP7 at the mRNA level (Additional file 3: Figure S3A), and BMP2 levels in the supernatants from sphere cells were higher than levels found in the cell culture supernatants from the monolayer cells (Additional file 3: Figure S3B). While levels of BMP2 production by the sphere cells decreased over a 14-day period, BMP2 levels remained higher, in general, than those found in the monolayer cell culture supernatants. Taken together, our results indicate that BMP signaling is reduced in sphere cells, and that this process is regulated, at least in part, by SMURF1. The insensitivity to the presence of BMP ligands provides evidence for CSC-like maintenance through BMP signaling inhibition, while increased BMP2 production likely indicates a paracrine growth promoting function of these cells.

### BMP signaling is decreased in CD44^high^/ALDH^high^ populations

**Figure 3:**
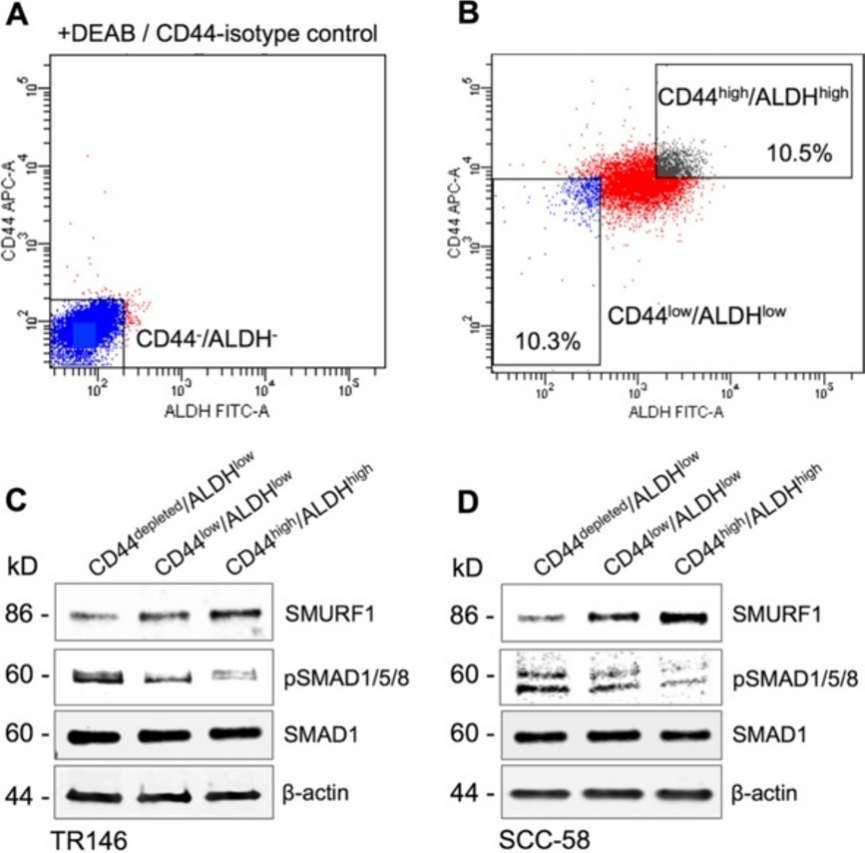
**BMP signaling and SMURF1 are differentially regulated in CD44** ^**high**^ **/ALDH** ^**high**^ **sorted CSC-like populations.** Representative FACS analyses of sorted cells are shown in A and B. **(A)** ALDEFLUOR stained cells in the presence of ALDH activity inhibitor DEAB and double-stained with APC-conjugated IgG isotype control. **(B)** Gating of CD44^low^/ALDH^low^ and CD44^high^/ALDH^high^ populations. **(C–D)** Immunoblotting of SMURF1, pSMAD1/5/8, and SMAD1 (as a control for total SMAD protein) in the sorted TR146 **(C)** and SCC-58 **(D)** cells shown as representatives of at least three repeats from two independent cell sorting experiments. CD44^+^ cells in the CD44^low^/ALDH^low^ sorted population were magnetically captured and removed to generate CD44^depleted^/ALDH^low^ cells. β-actin was used as a total protein loading control for all samples.

**Figure 4:**
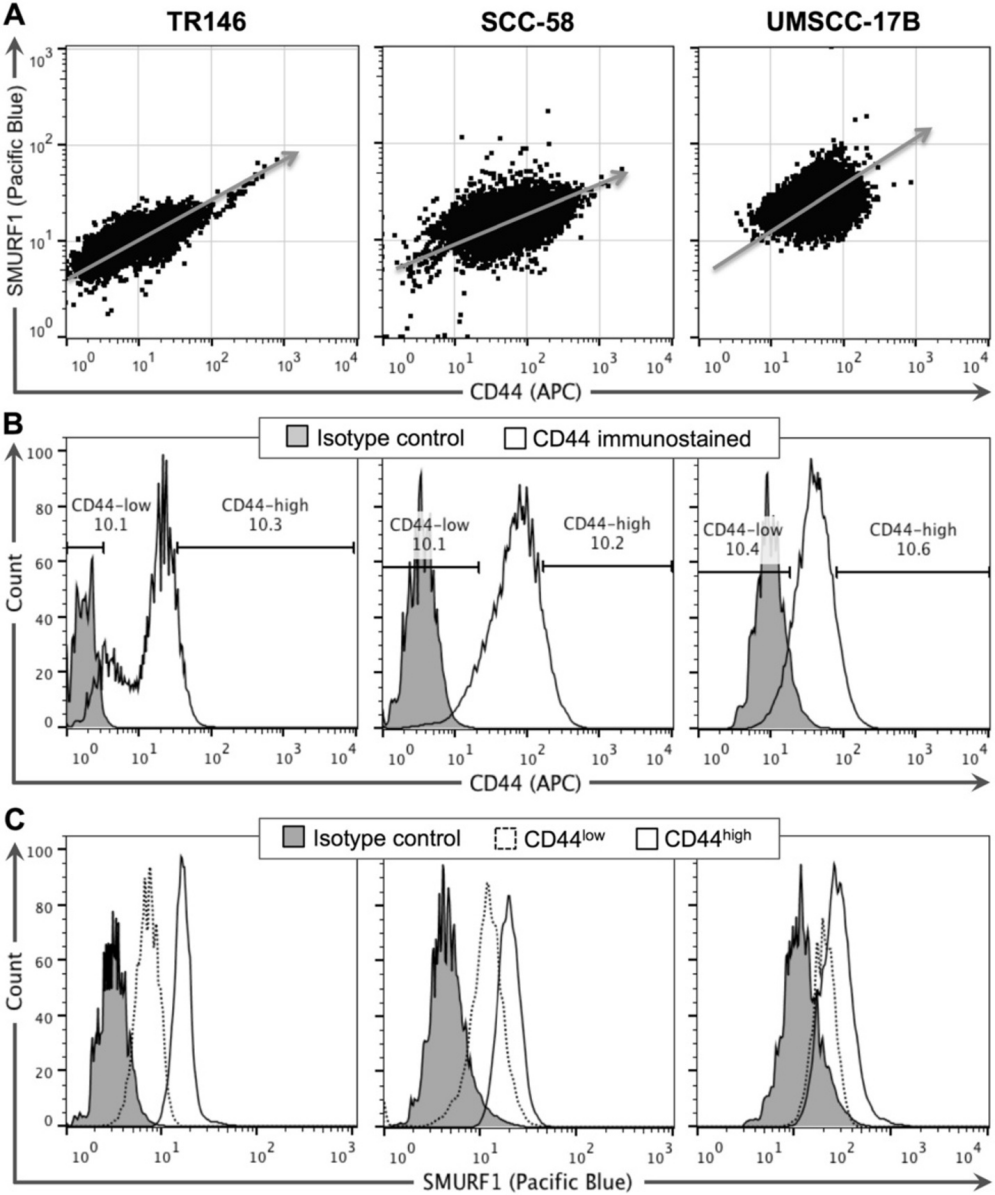
**SMURF1 is associated with CD44 surface expression in sphere-derived cells (SDCs). (A)** Flow cytometry analysis of SDCs showing a positive correlation (qualitatively represented by the grey arrow) between CD44 cell surface and intracellular SMURF1 protein expression. Cells were co-stained with fluorescently labeled anti-CD44 (APC) and anti-SMURF1 (Pacific Blue) and the total population is shown. Isotype staining control is shown in B. **(B)** Gating strategy used to define the CD44^high^ (top ~10%) and CD44^low^ (lower ~10%) SDC populations. **(C)** Histograms of SMURF1 expression in CD44^low^ and CD44^high^ populations based on the gates drawn in B. The data shown are representative of at least three repeats for each cell line (TR146, left column; SCC-58, middle column; UMSCC-17B, right column).

We next examined pSMAD1/5/8 levels in CD44^high^/ALDH^high^ cell populations to assess the activation status of BMP signaling since CD44^high^/ALDH^high^ cell populations have been shown to represent CSC-like populations in HNSCC and are highly tumorigenic *in vivo* [14, 20]. In addition, confirmation of decreased pSMAD1/5/8 levels in the CD44^high^/ALDH^high^ cells would further support the use of our sphere cell system to study the mechanism behind SMURF1 regulation of BMP signaling in HNSCC CSCs. TR146 and SCC-58 cells were cultured in standard monolayer conditions and stained for ALDH activity followed by CD44 cell surface immunostaining. Double stained cells were sorted based on the CD44^high^/ALDH^high^ and CD44^low^/ALDH^low^ markers (Figure 3A and B). The CD44^high^/ALDH^high^ cells showed increased SMURF1 protein levels while pSMAD1/5/8 protein levels were reduced compared to the CD44^low^/ALDH^low^ and CD44^depleted^/ALDH^low^ (cells further depleted for CD44) populations (Figure 3C and D). SMAD1 expression levels were unchanged. These results suggest that BMP signaling is regulated in a manner similar to that observed in the sphere cell populations. We then asked if CD44^high^ expression alone can serve as a marker for CSC-like cells in our study by performing a correlation analysis between CD44 and SMURF1. As expected, SMURF1 was highly associated with CD44 surface expression (Figure 4A). Flow cytometry analysis further indicated that SMURF1 expression was higher in CD44^high^ than in CD44^low^ cell populations (Figure 4B and C), demonstrating that higher SMURF1 levels are associated with cells reported to possess CSC-like properties. Based on these results, we sought to further define the role of SMURF1 in regulating BMP signaling.

### SMURF1 knockdown reactivates BMP signaling in CSC-like HNSCC cells

**Figure 5:**
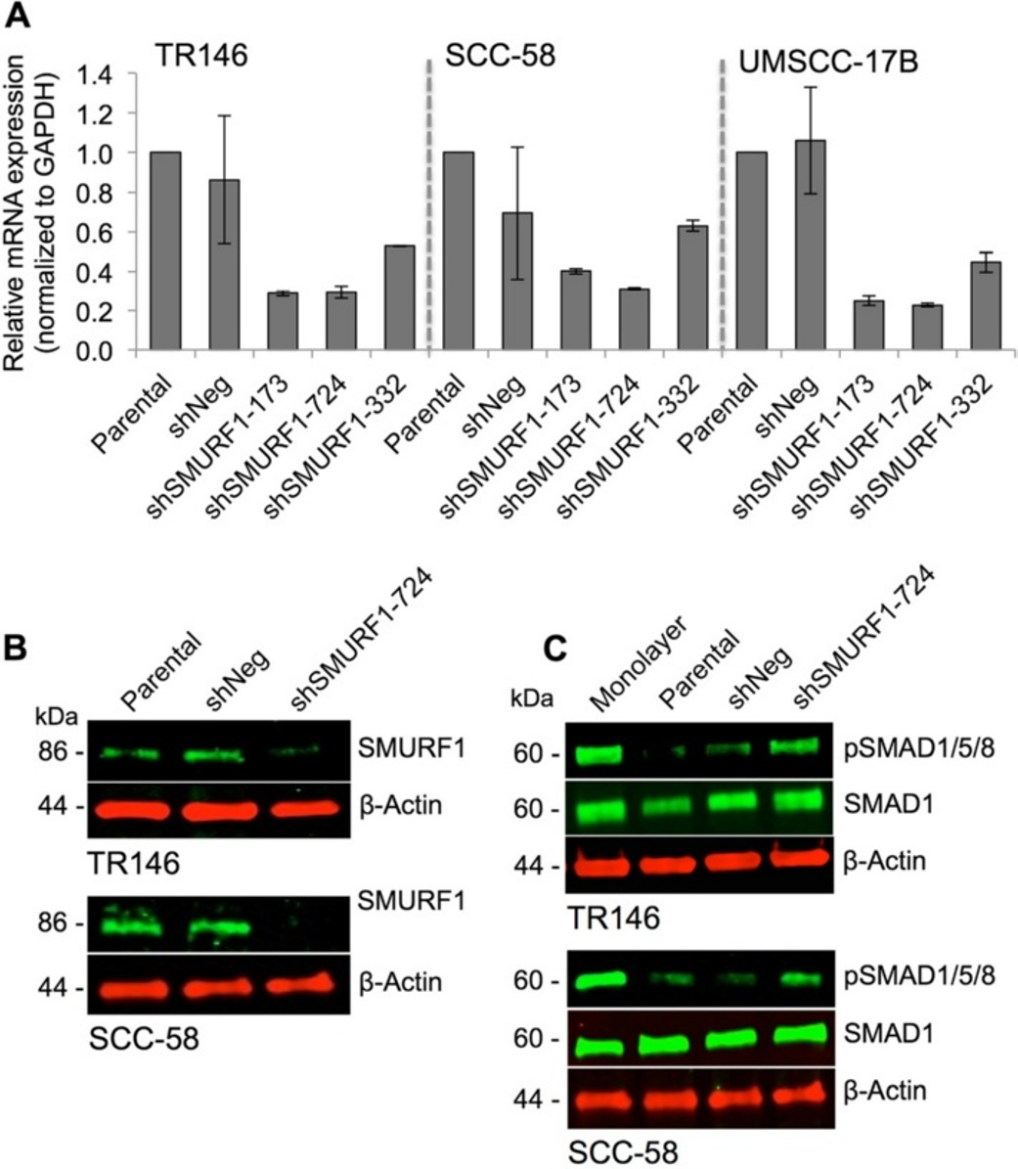
**SMURF1 knockdown reactivates BMP signaling in SDCs. (A)** SMURF1 knockdown screening by qRT-PCR in lentivirus transduced cells expressing negative knockdown shRNA control (shNeg) or one of three different shRNA constructs (shSMURF1-173, −724, and −332) targeting SMURF1 transcripts for silencing. Expression levels are relative to non-transduced parental control cells. Expression was normalized to the GAPDH housekeeping gene and presented as mean ± SD. Screening was performed in monolayer cells in duplicate. **(B)** SMURF1 immunoblotting in SDCs expressing shSMURF1-724 shRNA gene silencing construct relative to shNeg and parental controls from two cell lines. **(C)** Immunoblotting of pSMAD1/5/8 in SDCs from two cell lines expressing shSMURF1-724 shRNA gene silencing construct relative to shNeg, parental, and monolayer cells. β-actin was used for a gel loading control. Data shown are representative of at least three independent experiments.

Our data suggested that SMURF1 plays a role in the suppression of BMP signaling to maintain the CSC-like population. Although extracellular inhibitors may contribute to this inhibition, SMURF1 directly inhibits the activation of SMAD1, 5, and 8 and blocks the downstream BMP signaling cascade [44, 45]. To confirm this finding, we performed an expression knockdown study of SMURF1 using RNA interference. We transduced the monolayer cultures with either SMURF1 shRNA lentivirus (shSMURF1) or non-targeting shRNA (shNeg) with high efficiency (~90%) before placing the cells into cell culture conditions favoring sphere formation. Three different shRNA sequences were used to reduce SMURF1 expression, and the levels of SMURF1 mRNA were determined by qRT-PCR (Figure 5A). One sequence (shSMURF1-724) consistently reduced SMURF1 mRNA levels to the greatest extent in all cell lines. Decreased expression of SMURF1 was validated by immunoblotting (Figure 5B) in SDCs grown in the presence of FBS and Matrigel for 18–24 hours. Knockdown of SMURF1 led to partial reactivation of the BMP signaling pathway, indicated by the increase in pSMAD1/5/8 levels (Figure 5C). Taken together, our results show that SMURF1 regulates BMP signaling in CSC-like populations, which may have implications in maintaining the CSC-like phenotype.

### Inhibition of SMURF1 expression diminishes a subpopulation of CD44^high^ cells

**Figure 6:**
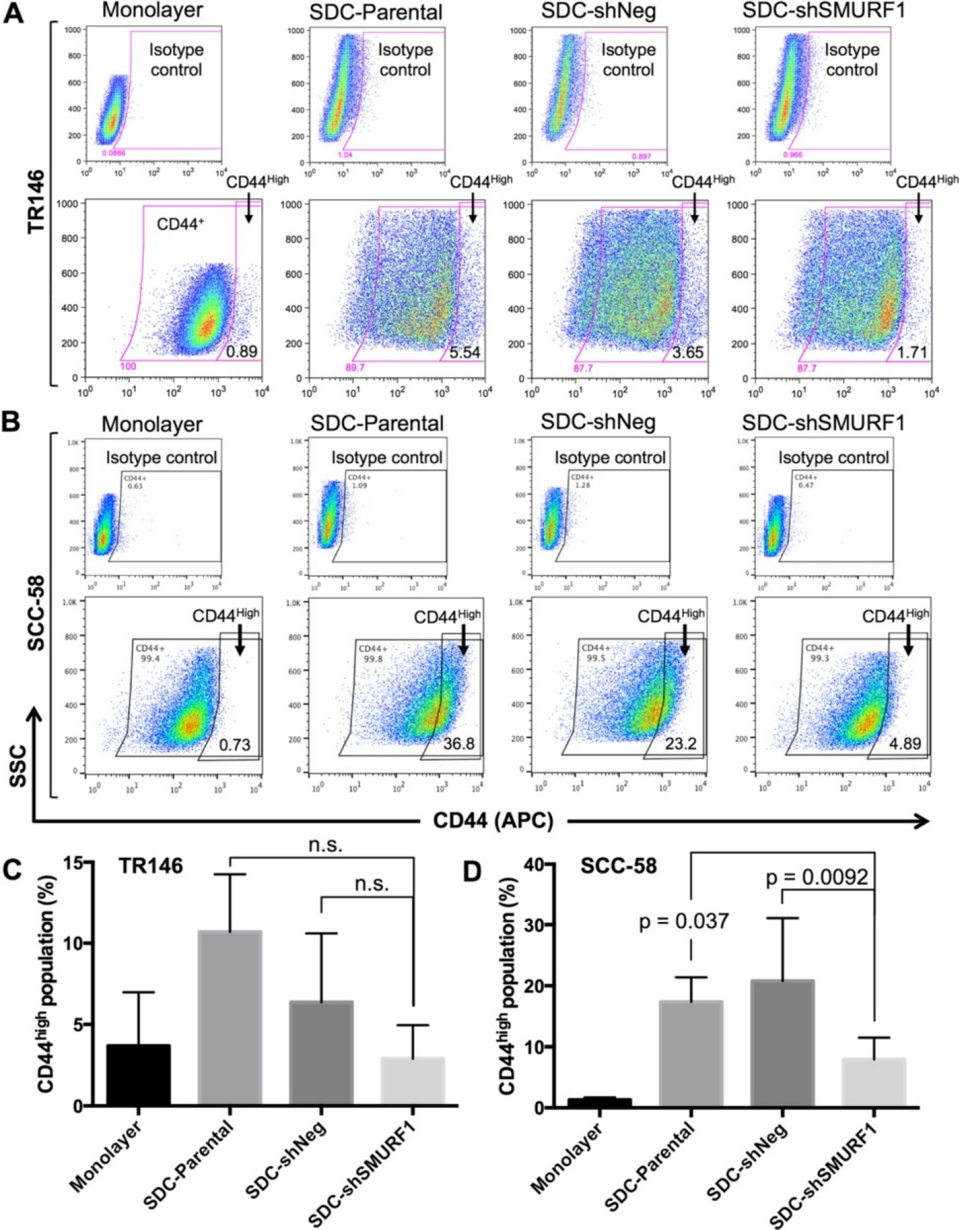
**SMURF1 inhibition reduces a CD44** ^**high**^ **cell population in SDCs.** Representative flow cytometry analysis of CD44^high^ cells in monolayer and in sphere-derived cells (SDCs) enriched from TR146 **(A)** and SCC-58 **(B)** sphere cultures are shown. A CD44^high^ population is reduced in SMURF1-knockdown (shSMURF1) compared to shNeg and parental control SDCs after culturing in complete medium for 4 – 11 days. **(C)** Summary of flow cytometry analysis of CD44^high^ TR146 SDCs shown in A (n = 5). **(D)** Summary of flow cytometry analysis of CD44^high^ SCC-58 SDCs shown in B (n = 7). Data in C and D are presented as mean ± SE for the indicated number of independent repeats. Statistical analysis was performed using two-tailed Student’s *t-test* with equal variance. n.s. = not significant (p > 0.05).

SMAD1 has been shown to interact directly with the intracellular domain of CD44 [22, 23, 46], and the E3 ligase activity of SMURF1 targets SMAD proteins for degradation [25, 47, 48]. Thus, SMURF1 provides a functional link between CD44 and the BMP signaling cascade. Based on the relative increase in expression of SMURF1 in CD44^high^ populations coupled with a decrease in BMP signaling, we hypothesized that SMURF1 plays a role in maintaining CSC-like cells within CD44^high^ cell populations. Therefore, decreasing SMURF1 expression could potentially reduce the levels of CSC-like cells through BMP activation and differentiation. Knockdown of SMURF1 expression decreased the percentage of CD44^high^ cells in SDC cultures grown for 4 – 11 days (Figure 6A and B), suggesting the loss of a specific subpopulation of CSC-like cells. The decrease in the CD44^high^ cells for TR146 SDCs following SMURF1 knockdown was highly reproducible in multiple, independent experiments (n = 5) but it was not statistically significant compared to the shNeg knockdown (p = 0.138) or the parental (p = 0.067) control cells (Figure 6C). This was likely due to variation in the parental and shNeg SDC CD44^high^ population enrichment from different experiments. Compared to the parental and shNeg controls, SMURF1 knockdown significantly reduced the number of CD44^high^ cells in SCC-58 SDCs (p = 0.0092 compared to shNeg, p = 0.037 compared to parental; n = 7) as shown in Figure 6D. We also noted that the CD44^high^ population in the TR146 SDCs was less than the CD44^high^ population in the SCC-58 SDCs, demonstrating the potential heterogeneity that may exist within HNSCC. Nonetheless, knockdown of SMURF1 greatly reduced the CD44^high^ population in both cases. Co-staining of CD44 and SMURF1 in SDCs further verified that the reduction of a CD44^high^ population correlated with the decreased expression of the SMURF1 protein (Additional file 4: Figure S4).

### Decreased expression of SMURF1 promotes differentiation and reduces tumorigenic capacity

**Figure 7:**
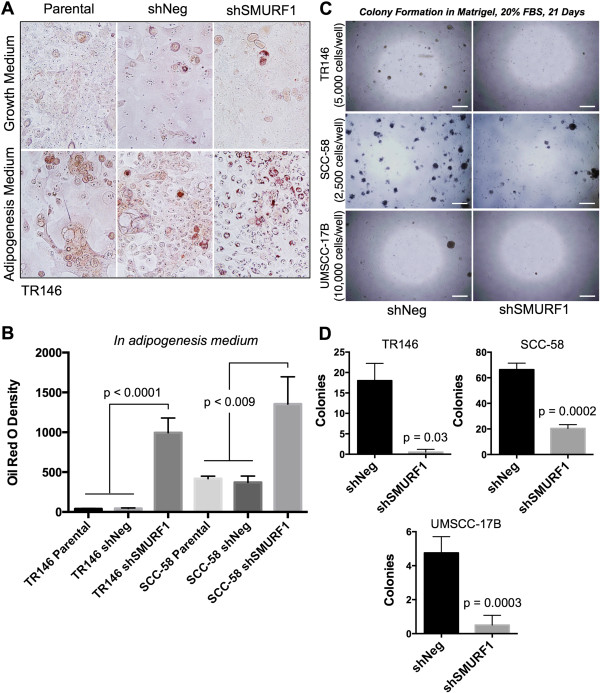
**SMURF1 knockdown promotes differentiation in SDCs and decreased colony formation by sphere cells. (A)** Adipogenesis culture as a measure for cellular differentiation. SDCs were grown in normal growth medium (top) or adipogenesis differentiation medium (bottom) for 7 days. Lipid droplet staining by Oil Red O was observed in SDCs with shSMURF1 knockdown grown in adipogenesis medium compared to shNeg or parental controls. Representative Oil Red O staining images shown are from the TR146 cell line. **(B)** Quantitation of Oil Red O staining in shNeg and shSMURF1 expressing SDCs cultured in adipogenesis medium from two representative cell lines. Data shown as mean ± SD, performed in triplicate experiments with multiple replicate wells. **(C)** Representative images of colony formation by SDCs in Matrigel anchorage-independent three-dimensional cultures supplemented with 20% FBS. **(D)** Summary of colony formation shown as mean number of colonies ± SE per 2,500 cells plated performed with three different cell lines as independent biological replicates (TR146, n = 2; SCC-58, n = 3; UMSCC-17B, n = 4). Statistical analysis was performed using two-tailed Student’s *t-test* with equal variance.

To confirm the loss of a CD44^high^ CSC-like population following SMURF1 knockdown, we tested whether these cells can be induced to undergo differentiation. To show differentiation capability, enriched CSC-like cells were cultured in adipogenesis differentiation medium for seven days. Lipid droplets were apparent in shSMURF1 SDCs after seven days in differentiating medium, but droplets were not present or not as prevalent in the parental and shNeg control SDCs or monolayer cells (Figure 7A). Both the parental and shNeg control SDCs showed similar background Oil Red O staining. Comparative analysis showed a significantly higher level (TR146, p < 0.0001; SCC-58, p < 0.009) of Oil Red O staining of lipid droplets in shSMURF1 SDCs than the shNeg or the parental SDCs (Figure 7B). These data suggest that sphere-enriched CSC-like cells have a progenitor-like phenotype that can be induced to differentiate by inhibiting SMURF1 and support the idea that SMURF1 plays a role in maintaining a CSC-like state.Finally, we tested the ability of the sphere cell populations to form colonies from single-cell suspension in three-dimensional (3D), anchorage-independent medium supplemented with 20% FBS. Clonogenicity in anchorage-independent growth medium is a method used to examine the tumor initiating capacity of cells. The control shNeg sphere cells formed colonies within 21 days, but shSMURF1 cells did not form colonies or formed a significantly lower number of colonies (TR146, p = 0.03; SCC-58, p = 0.0002; UMSCC-17B, p = 0.0003) (Figure 7C and D). These results indicate that shSMURF1 sphere cells may have lost their capacity for anchorage-independent survival and self-renewal, suggesting a loss in tumorigenic capacity.

## Discussion

Subpopulations of tumor initiating cells from HNSCC are currently defined by their high expression of CD44 and BMI1 along with their high ALDH activity [13, 14, 19, 38, 49, 50]. In addition, CD44^high^/ALDH^high^ cells are highly tumorigenic and recapitulate heterogeneous tumors in mice. While the epithelial-to-mesenchymal transition regulator Snail was found to be key in maintaining the CSC properties in HNSCC through its regulation of ALDH expression [12], little else is known about how these populations are modulated or maintained. Here, we expand this line of investigation by utilizing CD44^high^ expressing cells to represent CSC-like subpopulations from HNSCC cell lines combined with an analysis of the role of BMP signaling in regulating their maintenance. To do this, we enriched CSC-like cell populations, grown as non-adherent spheres, from three HNSCC cell lines. The sphere cells possessed CSC-like characteristics, including high expression of HNSCC CSC markers, CD44 and BMI1, resistance to the anti-cancer drug cisplatin, regeneration of spheres in suspension over multiple passages (self-renewal), and formation of visible colonies in three-dimensional culture assays. Sphere cells also showed increased expression of SMURF1 and inhibition of BMP signaling, indicating a potential regulatory mechanism for CSCs. The inverse relationship between SMURF1 and activation of the BMP signaling pathway was further confirmed in ALDH^high^/CD44^high^ populations, validating the use of our sphere cell culture system to test the hypothesis that BMP signaling regulates the fate of HNSCC CSCs.

BMP signaling is essential for cellular development, including induction of proliferation and differentiation, and has been shown to influence CSC populations in other cancers. In support of our hypothesis, activation of the BMP signaling cascade was shown to suppress CSC populations and metastatic potential of breast cancer [51], inhibit the tumorigenicity of CSCs in osteosarcoma and renal cell carcinoma [52, 53], and reverse CSC-like traits in intestinal adenoma [54] and brain tumor-initiating cells [55]. Furthermore, inhibition of BMP signaling enhanced the CSC-like traits and metastatic potential of breast cancer cells [56]. While these studies confirm that the inhibition of BMP signaling plays a role in maintaining progenitor cells in a CSC-like state, contradictory reports show that activation by BMP2 enhances CSC-like populations in ovarian carcinoma [57], and BMP4 stimulates an epithelial-to-mesenchymal and a CSC-like phenotype in a squamous cell carcinoma cell line of the tongue [37]. Thus, the BMP signaling mechanisms utilized by various cancers to maintain their CSC populations may be tumor type specific. Alternatively, other mechanisms may come into play, which override the regulatory factors that contribute to inhibition or activation of BMP signaling. As a result, studies need to be undertaken to further define the role of BMP signaling in maintaining or regulating CSC-like cells.

To this end, we predicted that activation of the canonical BMP signaling pathway would counteract CSC phenotypes by signaling CSCs to undergo differentiation, resulting in the loss of self-renewal and the overall CSC population. In support of this idea, we determined that HNSCC sphere cells deactivate BMP signaling intracellularly by upregulating SMURF1, leading to decreased levels of pSMAD1/5/8 and ID1, the canonical BMP receptor signal transducer [30, 58, 59] and BMP signaling target gene [26, 60], respectively. In both sphere cells and CD44^high^/ALDH^high^ sorted cells, pSMAD1/5/8 was decreased. Reduced levels of pSMAD1/5/8 and increased CD44^high^ expression correlated strongly with increases in SMURF1 protein levels, indicating SMURF1 associated suppression of BMP signaling and maintenance of the CSC-like state. Knocking down SMURF1 partially restored BMP signaling and enhanced the induction of CSC-like cells to undergo differentiation as shown using an adipogenesis assay. The ability to differentiate suggests a progenitor-like phenotype of the sphere-enriched cells. In addition, reactivation of BMP signaling through suppression of SMURF1 reduced clonogenicity of CSC-like cells as shown by their inability to form colonies in anchorage-independent, 3D culture supplemented with FBS. Because differentiation and loss of clonogenicity occurred only after downregulation of SMURF1 and reactivation of BMP pathway, these results support our hypothesis that SMURF1 inhibition of BMP signaling supports the maintenance of CSC-like tumor population.

SMURF1 targets receptor-regulated SMAD proteins, primarily SMAD1, 5 and 8, in the BMP signaling pathway for degradation through association with inhibitory SMAD6/7, or I-SMADs [43–45]. Through I-SMADs, SMURF1 also can induce ubiquitination and degradation of BMP type I receptors, inhibiting the BMP signaling cascade [61]. While pSMAD1/5/8 levels were reduced overall in the presence of increased expression of SMURF1 in our studies, decreases in SMAD1 expression were not detected, a surprising result since SMURF1 targets SMADs for degradation. It also is possible that partial degradation of the targeted SMADs by SMURF1 may be occurring, and changes were below the detection limit of our assays. While other methods will need to be employed in order address this possibility, our observations suggest that regulation of BMP signaling by SMURF1 is likely to be more complex than previously thought, and it may explain why BMP signaling was only partially restored by reduced expression of SMURF1 in our assays.

To add to this complexity, previous studies in chondrocytes have demonstrated a direct interaction between CD44 and SMAD1 [22, 23]. Here, CD44 acts as an intracellular scaffold protein to localize SMAD1 near BMP receptors, likely enhancing SMAD1 phosphorylation and activation [23]. Binding to CD44 in CSC-like cells may protect SMAD1 from degradation by SMURF1 but can also sequester the protein from phosphorylation and activation. How SMAD1 association with CD44 is regulated is not known. However, it is likely that the SMAD1/CD44 interaction provides a more precise and rapid response to BMPs as well as extracellular matrix proteins and conditions making the degradation of SMAD1 undesirable. It also is possible that partial release of SMAD1 from CD44 may occur upon loss of SMURF1 or limited activation of the BMP receptor. Thus, the role of SMAD1/CD44 signaling in the context of HNSCC CSCs warrants further investigation.

While our data are the first to implicate SMURF1 in the regulation of a CSC-like population, SMURF1 may play a similar role in other cancers. In human colorectal cancer, elevated expression of SMURF1 has been shown to correlate with cancer progression and prognosis [62]. In pancreatic cancer, SMURF1 amplification promotes tumor invasiveness [41], suggesting that SMURF1 might be used as an indicator of progression and/or prognosis for some cancers including those of the head and neck. While we have not yet undertaken such a study, the expression levels of SMURF1 in primary, recurrent, and drug resistant HNSCC tumors may be informative.

The recognition that other ubiquitin ligases play key roles in regulating both stem cells and CSC populations is becoming more widespread. The F-box protein Fbxw7, which is the substrate-recognition subunit of an SCF-type ubiquitin ligase complex, interacts with and mediates the ubiquitylation of c-Myc [63]. The ubiquitin-dependent degradation of c-Myc has been found to be essential for maintenance of the quiescence and reconstitution capacity of normal hematopoietic stem cells. For CSCs, Takeishi et al. [64] showed that Fbxw7 plays a pivotal role in maintenance of quiescence in leukemia initiating cells (LICs) in chronic myeloid leukemia by reducing the level of c-Myc. Abrogation of quiescence in LICs by Fbxw7 ablation increased LIC sensitivity to the tyrosine kinase inhibitor imatinib, and the combination of Fbxw7 ablation with imatinib treatment resulted in a greater depletion of LICs. Furthermore, King et al. [65] demonstrated that mutations in Fbxw7 specifically enhanced cancer-initiating cell activity in collaboration with Notch1 oncogenes in a model of T-cell acute lymphoblastic leukemia but spared normal hematopoietic stem cell function. Finally, the E3 ligase, Skp2, has been shown to be overexpressed in numerous human cancers and plays a critical role in cell-cycle progression, senescence, metabolism, cancer progression, and metastasis. By using a Skp2 inhibitor, Chan et al. showed Skp2 positively regulates CSC populations and self-renewal ability [66]. Notably, the Skp2 inhibitor exhibited potent antitumor activities in multiple animal models and cooperated with chemotherapeutic agents to reduce cancer cell survival. Based on these and other studies, ubiquitin ligases are promising targets for restricting cancer stem cell populations and cancer progression. In this regard, small molecule compounds that block the WW1 domain of Smurf1 from interacting with Smad1/5 have been reported in mouse cell lines [67], pointing to the feasibility of targeting human SMURF1 for therapeutic intervention.

## Conclusions

Our findings suggest that SMURF1 inhibition of BMP signaling in CSC-like populations potentiates the long-term survival and maintenance of CSCs in head and neck cancer. Increased expression of SMURF1 and suppressed BMP signaling were found in sphere cell cultures and CD44^high^/ALDH^high^ sorted populations. Restoration of BMP signaling by silencing SMURF1 reactivated BMP signaling, leading to increased cellular differentiation and reduction in the CD44^high^ CSC phenotype. Our data strongly suggest a loss in self-renewal and tumorigenicity of CSC-like cells following SMURF1 knockdown. Thus, SMURF1 represents a potential drug target to inhibit the maintenance and progression of CSCs.

## Methods

### Cell lines and cell culture conditions

The HNSCC cell lines TR146, SCC-58, and UMSCC-17B were from the laboratory of Dr. Mark Herzberg, and their sources of origin and culture conditions were described previously [68]. Cells were cultured and maintained on standard tissue-culture treated flasks in Dulbecco’s Modified Eagle Medium/Ham’s F-12 50:50 mix (DMEM/F-12) supplemented with 10% fetal bovine serum (FBS) and 0.2% Primocin™ (InvivoGen, San Diego, CA, USA). The cells were maintained in 5% CO_2_ at 37°C. Each cell line was tested periodically for *Mycoplasma* expression by qPCR.

### Sphere cell culture

Sphere cells were cultured as described previously but with the following modifications [10, 34, 35]. Monolayer cells were harvested by trypsinization when the flasks were approximately 75% confluent and cultured in DMEM/F-12 supplemented with 0.4% bovine serum albumin or BSA, 10 ng/mL b-FGF, 20 ng/mL EGF, and 5 μg/mL insulin. Cells were cultured as single-cell suspensions in ultralow attachment culture flasks (Corning Inc., Corning, NY) for 10 to 14 days. Spheres were collected every 3 – 4 days by gravity to remove dead and non-sphere forming cells in the supernatant. The spheres were washed with PBS, and separated into single cell suspensions by incubating with 1 mL of 0.25% trypsin-EDTA for 2 – 4 min at room temperature. To generate sphere-derived cells (SDCs) for short-term proliferative growth studies, single cell suspensions of sphere cells were cultured on adherent tissue-culture treated flasks in complete medium supplemented with 75 μg/mL Matrigel™ Basement Membrane Matrix Growth Factor Reduced (Stemcell Technologies Inc., Vancouver, BC, Canada).

### Cell sorting for CD44^high^/ALDH^high^ cells

Monolayer cultured cells (15–20 ×10^6^) were harvested at approximately 70% confluency and stained for ALDH activity using an ALDEFLUOR™ Kit (Stemcell Technologies Inc.) based on the manufacturer’s protocol. Following ALDEFLUOR staining, cells were centrifuged and resuspended in ice-cold staining buffer (ALDEFLUOR buffer containing 2% FBS). Allophycocyanin (APC) conjugated rat monoclonal antibody against human CD44 (clone IM7; eBioscience Inc., San Diego, CA) was added at a 1:100 dilution and incubated on ice for 30 min in the dark. APC conjugated rat IgG2b isotype (eBioscience Inc.) was used as a negative staining control. Cells were washed with 10x volume of staining buffer and the cell number adjusted to 1 × 10^7^ cells/mL with staining buffer for sorting. Fluorescence activated cell sorting (FACS) was performed using a BD FACSAria II (BD Biosciences, San Jose, CA) equipped with a 100 μm nozzle. CD44^high^/ALDH^high^ cells were sorted for by gating from the top 10% of double APC/FITC positive cells. CD44^low^/ALDH^low/-^ cells were selected by gating for the lower 10 – 20% of the APC^low^/FITC^low/-^ cells. To further deplete CD44 positive cells, the sorted cells were expanded under standard monolayer culture conditions for four days, followed by CD44-positive cell depletion using an EasySep APC selection kit (Stemcell Technologies Inc.). Cultured CD44^low^/ALDH^low/-^ sorted cells were stained with APC conjugated anti-CD44 antibody, washed and magnetically captured and removed. Flow-through cell suspension was collected through magnetic separation several times, generating a population of CD44^depleted^/ALDH^low^ cells.

### Gene expression analysis by qRT-PCR and PCR array

Gene expression analysis by real-time quantitative RT-PCR (qRT-PCR) was performed using standard techniques. Total RNA extraction from cultured cells was performed using an RNeasy Mini Kit (Qiagen Inc., Valencia, CA) and analyzed using a Nanodrop spectrophotometer (Thermo Fisher Scientific Inc., Wilmington, DE). Samples were reverse transcribed using a SuperScript® VILO cDNA synthesis kit (Life Technologies, Grand Island, NY). Real-time PCR was performed using FastStart Universal SYBR Green master mix (Roche Diagnostics GmbH, Mannheim, Germany) based on the manufacturer’s protocol. Primers used for qRT-PCR were:

*CD44*

5′-CATCTACCCCAGCAACCCTA-3′ (sense)

5′-ATCCCAGGTTTCTTGCCTCT-3′ (antisense)

*BMI1*

5′-CAGCAATGACTGTGATGCACT-3′ (sense)

5′-GGACCATTCCTTCTCCAGGT-3′ (antisense)

*CD24*

5′-ACCCACGCAGATTTATTCCA-3′ (sense)

5′-ACCACGAAGAGACTGGCTGT-3′ (antisense)

*SMURF1*

5′-CTGGATGCTTTTGGTCTGGT-3′ (sense)

5′-CCTGATAGACGCGAACACAG-3′ (antisense)

*BMPER*

5′-ACATCGACCTGGATGGCTAC-3′ (sense)

5′-CCCTCCAAGATTCAGCAAAG-3′ (antisense)

*BAMBI*

5′-ATCGCCACTCCAGCTACATC-3′ (sense)

5′-TGTCGTGCTTGCAAGAGAGT-3′ (antisense)

*ID1*

5′-CTCCAGCACGTCATCGACTA-3′ (sense)

5′-CGCTTCAGCGACACAAGAT-3′ (antisense)

*ID2*

5′-CCTCAACACGGATATCAGCA-3′ (sense)

5′-CCTCCTTGTGAAATGGTTGAA-3′ (antisense)

BMP signaling pathway gene expression analysis was performed using a human TGFβ/BMP Signaling Pathway RT^2^ Profiler PCR Array System (Qiagen Inc.) with the provided RT^2^ First Strand Kit for cDNA synthesis, preloaded primer sets in 96-well format, and RT^2^ SYBR Green/ROX PCR Master mix, according to manufacturer’s protocol. PCR reactions were performed in Mastercycler® ep *realplex 2* (Eppendorf, Hauppauge, NY) real-time thermal cycler.

### SMURF1 knockdown by lentivirus delivered shRNA system

Human SMURF1 short hairpin RNA (shRNA) plasmids were purchased from Open Biosystems (Thermo Fisher Scientific Inc.). Three different SMURF1 targeting and silencing plasmids containing green fluorescence protein (GFP) and puromycin resistant genes (V2LHS_229724, V2LHS_203332, and V2LHS_363173) were supplied in *E. coli* competent cells. The cultures were expanded and the plasmids extracted using a QIAGEN Plasmid Midi kit (Qiagen Inc.). Extracted plasmids were then packaged into a Trans-Lentiviral™ GIPZ lentivirus packaging system (Open Biosystems) and transfected into the TLA-HEK293T producer cell line according to the manufacturer’s supplied protocol. Viral particle supernatants were collected at 48 and 72 h post-transfection and clarified by centrifugation. Clarified supernatants were filtered through a sterile 0.45 μm low protein-binding filter and concentrated to 1/100^th^ of the original volume using Lenti-X Concentrator reagent (Clontech Laboratories, Inc., Mountain View, CA). Concentrated viral particles were used to transduce cells in adherent monolayer culture at approximately 50% confluency in 1 mL complete medium supplemented with 8 μg/mL polybrene in 6-well plates overnight. After overnight incubation, viral particles were removed and the medium changed to normal growth conditions. Cells expressing either non-targeting random sequence shRNA (shNeg) control vector or SMURF1 targeting shRNA (shSMURF1) were monitored for GFP expression and further grown in selective medium containing 2 μg/mL puromycin for 72 h to eliminate non-transduced cells.

### Protein extraction

Total protein was extracted from approximately 1 × 10^6^ cells by incubating in RIPA lysis buffer (150 nM sodium chloride, 1.0% Triton X-100, 0.5% sodium deoxycholate, 0.1% sodium dodecyl sulphate, 50 nM Tris, pH 8.0, 10% glycerol) supplemented with fresh Halt™ protease and phosphatase inhibitor cocktail (Thermo Fisher Scientific Inc.) at 1:100 dilutions for 30 min on ice with periodic vortexing. Protein lysate supernatant was collected by centrifugation at 20,000 × g for 10 min at 4°C to remove insoluble materials, and the protein concentrations determined using a BCA™ protein assay kit (Thermo Fisher Scientific Inc.).

### Immunoblotting analysis

Immunoblotting was performed based on standard techniques as described previously [68]. Briefly, protein samples were boiled in 1X sample buffer and loaded onto 4 – 15% gradient SDS-polyacrylamide gel at 50 μg/well for electrophoresis, transferred to nitrocellulose membrane, and blocked with 50% Odyssey® Blocking Buffer (LI-COR Biosciences, Lincoln, NE) diluted in TBST (20 mM Tris–HCl pH 7.4, 137 mM NaCl, 0.1% Tween-20). Rabbit anti-SMURF1 primary polyclonal antibody (Cat# AB10005; EMD Millipore, Billerica, MA) was added at a 1:300 dilution in blocking buffer and incubated for 1 h at room temperature. Rabbit anti-phospho-SMAD1 (Ser463/465)/SMAD5 (Ser463/465)/SMAD8 (Ser426/428) or pSMAD1/5/8 (Cat# 9511; Cell Signaling Technology, Danvers, MA) and rabbit anti-SMAD1 as a total SMAD protein control (Cat# 6944; Cell Signaling Technology) were used at a 1:1000 dilution. Mouse anti-β-actin antibody (1:1000 dilution) was used as loading control. The membrane was washed three times with TBST, followed by incubation with LI-COR IRDye 800CW (780 nm) donkey anti-rabbit and IRDye 680RD (680 nm) donkey anti-mouse infrared fluorescence dye conjugated secondary antibodies (LI-COR Biosciences) at a 1:10,000 dilution for 10 – 15 min at room temperature using the SNAP i.d. Western Blotting System (EMD Millipore). After washing to remove excess secondary antibodies and Tween-20, the membranes were scanned and documented using an Odyssey infrared imaging system (LI-COR Biosciences) at 680 nm and 780 nm emission wavelengths.

### Immunostaining and flow cytometry

Cell surface protein immunostaining was performed on single-cell suspensions of live cells. Approximately 1 × 10^6^ cells were incubated in immunostaining buffer (PBS containing 2% FBS and 2 mM EDTA) for 10 min on ice followed by incubation with the fluorescence conjugated primary antibody on ice, for 30 min, and in the dark. APC conjugated rat anti-human CD44 monoclonal antibody IgG2bκ was used as described above at a 1:100 dilution to detect CD44-positive cells. An APC conjugated rat non-specific IgG2b isotype was used as a negative staining control. For co-staining with mouse monoclonal antibody to detect intracellular SMURF1 protein, cells stained with CD44-APC antibody were fixed in 10% phosphate buffered formalin for 30 min on ice, permeabilized with ice-cold 100% methanol for 30 min on ice or overnight at 4°C, washed three times with staining buffer, and incubated with fluorescently labeled mouse anti-human SMURF1 monoclonal antibody (Sigma-Aldrich, Saint Louis, MO) at 1 μg per 1 × 10^6^ cells in PBS plus 1% BSA on ice for 30 min. Anti-SMURF1 antibody was labeled with Zenon® Pacific Blue fluorochrome for mouse IgG2a (Life Technologies). Mouse non-specific IgG2a isotype was labeled similarly and used as a negative staining control. The cells were washed, resuspended in staining buffer, and analyzed using a BD LSR II flow cytometer (BD Biosciences).

### Adipogenesis differentiation assay with Oil Red O staining

Adipogenesis assays were carried out using a StemPro® Adipogenesis Differentiation kit (Life Technologies). Monolayer and sphere cells were cultured overnight in complete medium before switching to differentiation medium. Complete medium and sphere cell culture medium were used as negative controls for the monolayer and sphere cells, respectively. Cells were grown in differentiation or control medium for 7–14 days, with medium change every three days. To visualize intracellular microlipid droplets, growth media were carefully aspirated and cells were fixed in 4% paraformaldehyde for 30 – 40 min at room temperature. Following three washes with PBS and twice with water, Oil Red O solution (EMD Millipore) was added to cover the cells and incubated at room temperature for 50 min. Cells were then washed three times with water, visualized under light microscopy, and imaged.

### Anchorage-independent colony formation

Cells (~5,000-10,000 per well) were grown as single-cell suspensions in complete medium containing 20% FBS and 50% Matrigel™ matrix on Ultra Low Cluster 96-well plates (Corning, Inc.). Embedded cells were incubated in 37°C, 5% CO_2_ overnight to allow matrix polymerization before topping off each well with 200 μL of complete medium. Cells were fed every seven days for a total of 21 days to allow colonies to form. Colonies were visualized by light microscopy using the lowest (4X) objective.

### Cytotoxicity, cell viability, and cisplatin resistance

Cellular cytotoxicity assays were performed in tissue-culture treated 96-well plates. Cells were seeded at 5,000 – 10,000 cells per well, overnight. Serial dilutions of cisplatin (0 to 10 μM) were added to the cells and incubated for 72 h. Cell viability following cisplatin treatment was determined by CellTiter 96® MTS assay (Promega Corp., Madison, WI). Cells were incubated with MTS solution for 3 h at 37°C, 5% CO_2,_ and the absorbance was measured at 490 nm. Percent viability was calculated relative to untreated control.

### Statistics

Statistical analysis was performed using unpaired two-tailed Student’s *t-test* with equal variance. Comparative analysis with p-value < 0.05 was considered significant.

## Electronic supplementary material

Additional file 1: Figure S1: Representative flow cytometry analysis of CD44 surface protein expression in **(A)** TR146, **(B)** SCC-58, and **(C)** UMSCC-17B monolayer (top) and sphere (bottom) cells as summarized in Figure 1C. Isotype controls are shown on the left in each panel. (JPEG 2 MB)

Additional file 2: Figure S2: Sphere cells are more resistant to cisplatin than monolayer cells. Representative results from the SCC-58 and TR146 cell lines are shown. Cells were treated with cisplatin for 72 h and ratios of viable cells were measured by an MTS assay. Data are shown as mean ± SD from at least three independent experiments. (JPEG 693 KB)

Additional file 3: Figure S3: BMP ligand expression and secretion by sphere cells. **(A)** BMP2, 4, and 7 expression in sphere cells relative to monolayer cultures by qRT-PCR, shown as a representative of two repeats. **(B)** BMP2 secretion was higher in sphere cell cultures compared to their monolayer counterparts. Cell culture supernatants were collected at day 3 from the monolayer cells and at days 3, 7, and 14 from the sphere cells with a fresh medium change one day prior to sampling. The level of extracellular BMP2 production was measured using an ELISA and protein concentrations were normalized to the level of viable cells present based on MTS absorbance values. Data are presented as the concentration of BMP2 per MTS absorbance value, mean ± SD, performed in duplicate. (JPEG 917 KB)

Additional file 4: Figure S4: Representative flow cytometry analysis of SDCs double-stained with CD44 cell surface and intracellular SMURF1 proteins. The data shown are representative of at least three independent experiments. (JPEG 1005 KB)
