# Supplementary figures and images for: SMURF1 silencing diminishes a CD44-high cancer stem cell-like population in head and neck squamous cell carcinoma
Source: Mol Cancer. 2014 Dec 3;13:260. doi: 10.1186/1476-4598-13-260 (PMC4265428; doi:10.1186/1476-4598-13-260)

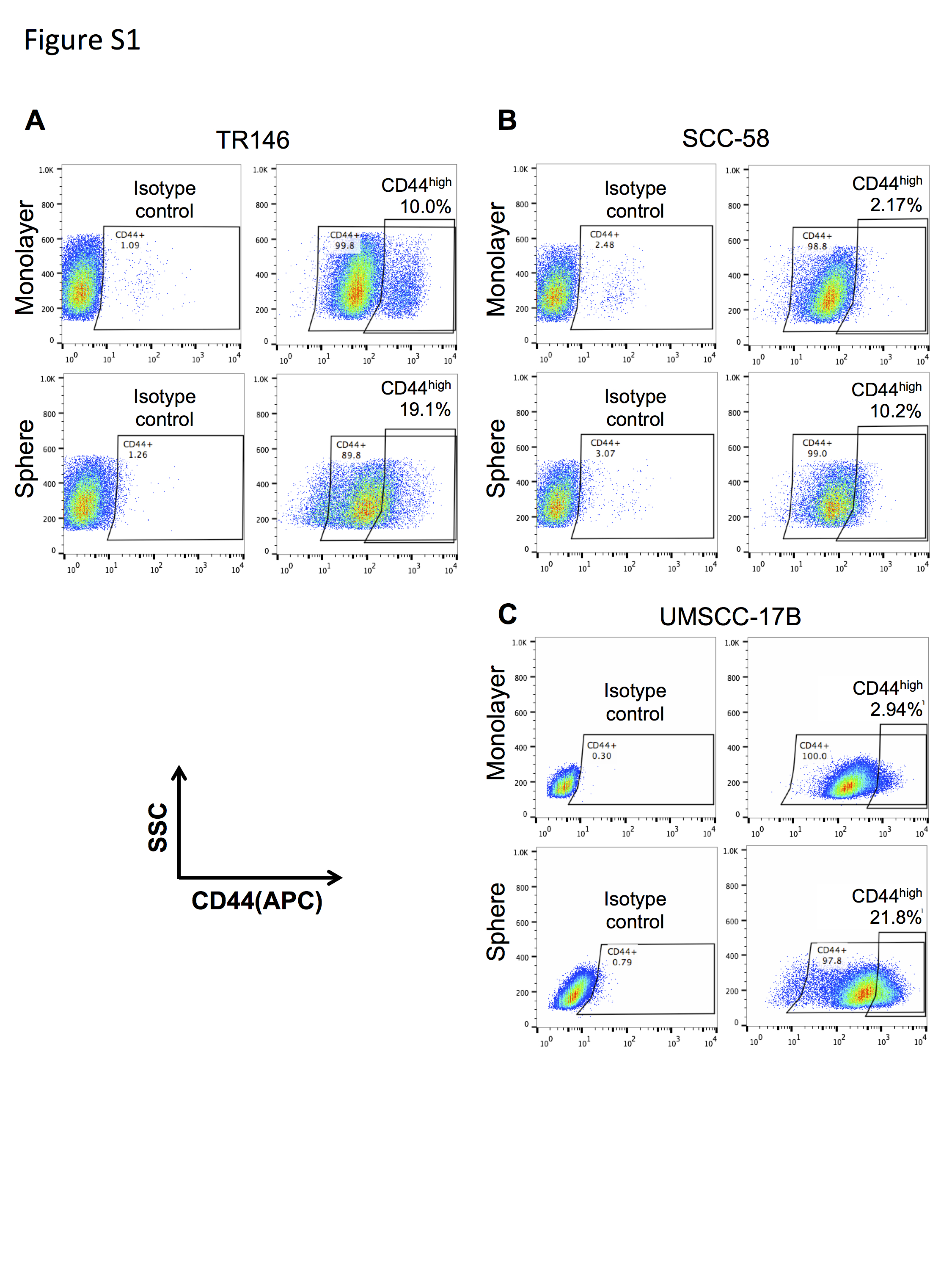

Supplement: Supplementary file 1 — Additional file 1: Figure S1: Representative flow cytometry analysis of CD44 surface protein expression in (A) TR146, (B) SCC-58, and (C) UMSCC-17B monolayer (top) and sphere (bottom) cells as summarized in Figure 1C. Isotype controls are shown on the left in each panel. (JPEG 2 MB) [file 12943_2014_1456_MOESM1_ESM.jpeg]

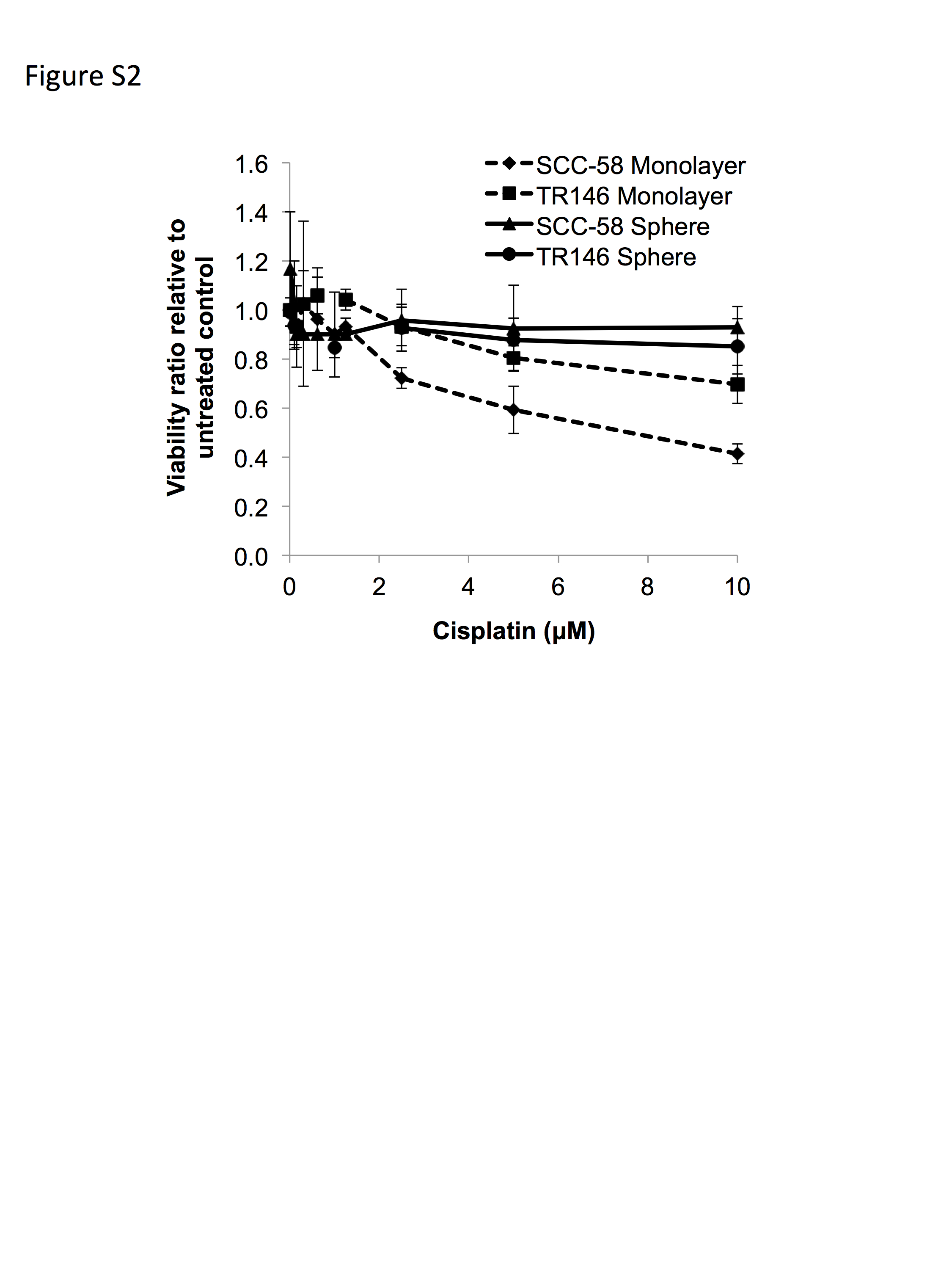

Supplement: Supplementary file 2 — Additional file 2: Figure S2: Sphere cells are more resistant to cisplatin than monolayer cells. Representative results from the SCC-58 and TR146 cell lines are shown. Cells were treated with cisplatin for 72 h and ratios of viable cells were measured by an MTS assay. Data are shown as mean ± SD from at least three independent experiments. (JPEG 693 KB) [file 12943_2014_1456_MOESM2_ESM.jpeg]

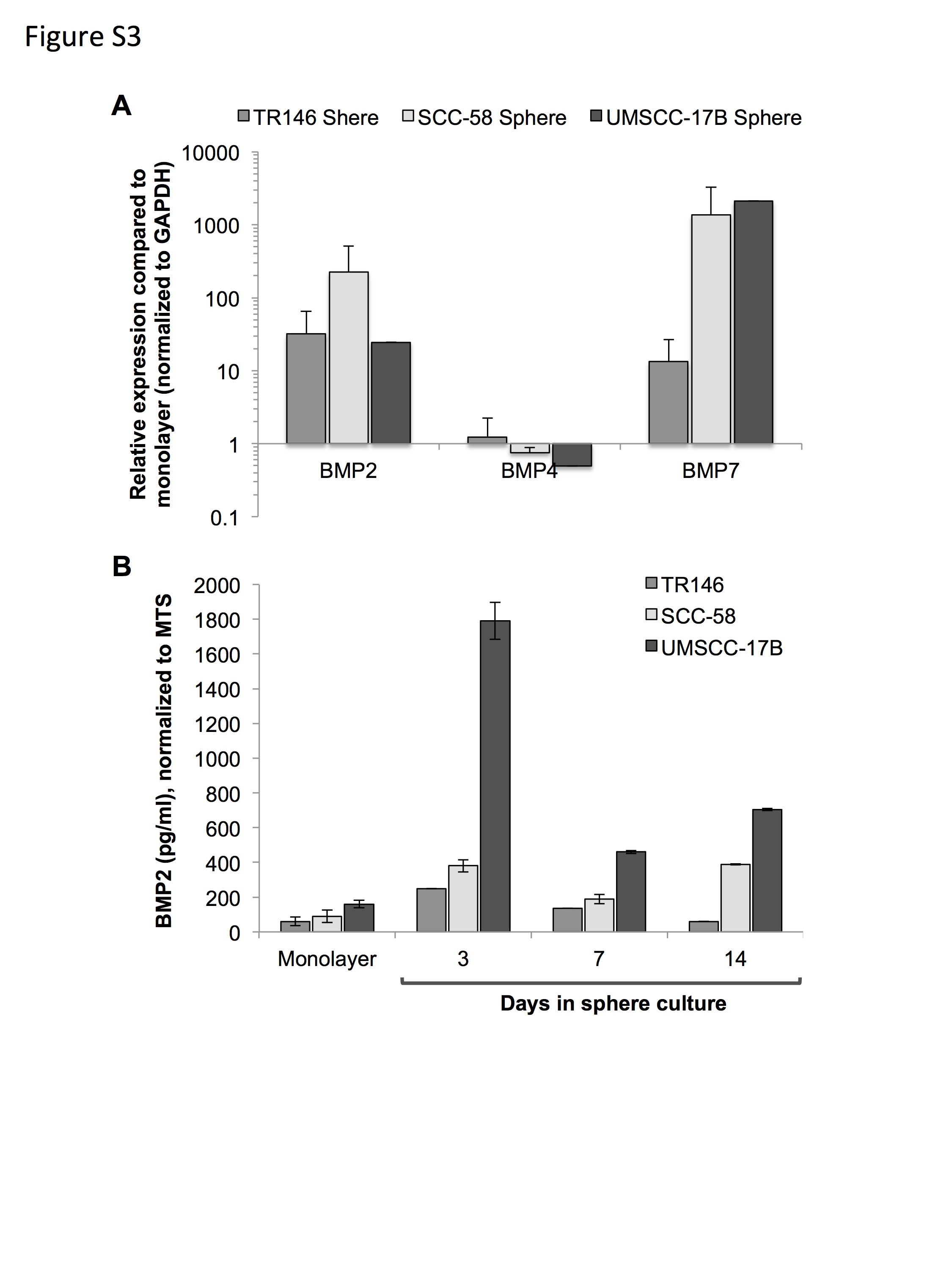

Supplement: Supplementary file 3 — Additional file 3: Figure S3: BMP ligand expression and secretion by sphere cells. (A) BMP2, 4, and 7 expression in sphere cells relative to monolayer cultures by qRT-PCR, shown as a representative of two repeats. (B) BMP2 secretion was higher in sphere cell cultures compared to their monolayer counterparts. Cell culture supernatants were collected at day 3 from the monolayer cells and at days 3, 7, and 14 from the sphere cells with a fresh medium change one day prior to sampling. The level of extracellular BMP2 production was measured using an ELISA and protein concentrations were normalized to the level of viable cells present based on MTS absorbance values. Data are presented as the concentration of BMP2 per MTS absorbance value, mean ± SD, performed in duplicate. (JPEG 917 KB) [file 12943_2014_1456_MOESM3_ESM.jpeg]

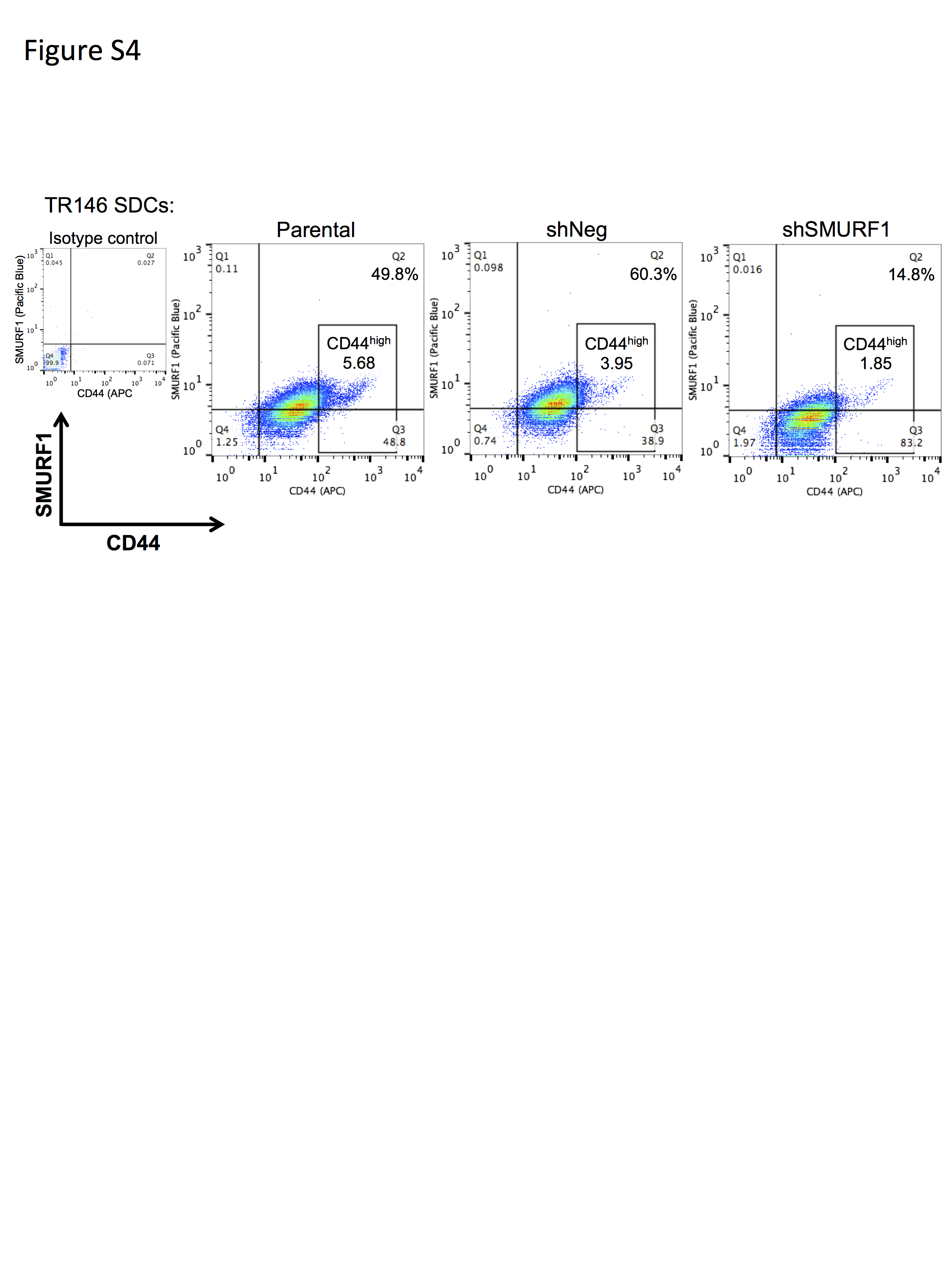

Supplement: Supplementary file 4 — Additional file 4: Figure S4: Representative flow cytometry analysis of SDCs double-stained with CD44 cell surface and intracellular SMURF1 proteins. The data shown are representative of at least three independent experiments. (JPEG 1005 KB) [file 12943_2014_1456_MOESM4_ESM.jpeg]
